# First description on female and tadpole of *Nidirana chongqingensis* Ma & Wang, 2023 (Anura, Ranidae) and its distribution expansion in Guizhou Province, China

**DOI:** 10.3897/BDJ.14.e183994

**Published:** 2026-02-06

**Authors:** Jing Liu, Yanlin Cheng, Lang Mu, Junjian Zhou, Jiantao Pan, Shize Li, Gang Wei

**Affiliations:** 1 College of Resources and Environment, Moutai Institute, Renhuai, China College of Resources and Environment, Moutai Institute Renhuai China; 2 College of Forestry, Guizhou University, Guiyang, China College of Forestry, Guizhou University Guiyang China; 3 Biodiversity Conservation Key Laboratory, Guiyang College, Guiyang, China Biodiversity Conservation Key Laboratory, Guiyang College Guiyang China

**Keywords:** *Nidirana chongqingensis*, morphology, phylogeny, tadpoles, Guizhou

## Abstract

**Background:**

The *Nidirana* Dubois, 1992 is widely distributed in East and Southeast Asia. In recent years, the species of this genus have been continuously updated, with 22 species recognised globally to date and 21 species recorded in China. *Nidirana chongqingensis* is a species described in 2023, previously only recorded from Qianjiang, Chongqing, China. With the deepening of surveys, this species has also been discovered in Zheng'an County, Guizhou Province, China, and its females and tadpoles have been first recorded herein.

**New information:**

During herpetological surveys in 2024–2025, we collected six adult specimens (3 males, 3 females) and five tadpoles of Nidirana from Zheng'an County, Guizhou Province, China. Molecular analyses of 16S and COI genes confirmed their identity as *N. chongqingensis*. We provide the first detailed description of the female morphology and tadpole characteristics of this species, revise its diagnostic features, extend its known distribution range from Chongqing to Guizhou, and offer foundational data for future conservation assessments.

## Introduction

The genus *Nidirana* (Ranidae) inhabits subtropical and tropical regions of East and Southeast Asia, ranging from Japan southwards to China, northern Thailand, Vietnam, and Laos (Fei et al. 2009, Lyu et al. 2017). Taxonomic classifications of *Nidirana* have long been contentious, with the genus previously treated as a subgenus or synonym of Rana Linnaeus, 1758, or merged with Babina Thompson, 1912 (Frost 2023). Molecular, morphological, and bioacoustic evidence later confirmed *Nidirana* as an independent genus (Lyu et al. 2017). In recent years, intensive surveys have revealed high cryptic diversity within the genus, with over 11 new species described from China since 2020(Lyu et al. 2020a, Lyu et al. 2020b, Wei et al. 2020, Lyu et al. 2021, Chen et al. 2022a, Chen et al. 2022b, Ma and Wang 2023, Lin et al. 2025, Liu et al. 2025) .

*Nidirana chongqingensis* was described by Ma and Wang (2023) based on three adult male specimens from Qianjiang District, Chongqing Municipality, China. The species is distinguishable from congeners by a combination of small male body size (SVL 41.8–43.3 mm), lateroventral grooves on fingers and toes, and specific webbing formula (Ma and Wang 2023). However, the lack of female and tadpole descriptions, as well as limited distribution data, hindered comprehensive understanding of its biology and conservation status.

During field surveys in Zheng'an County, Guizhou Province, China, in July 2024 and April 2025, we collected *Nidirana* specimens that matched the morphological diagnosis of *N. chongqingensis*. Molecular and detailed morphological analyses confirmed their identity. Herein, we present the first description of the female and tadpole of *N. chongqingensis*, revise its diagnostic characters, update its distribution, and discuss conservation implications.

## Materials and methods

**Sampling**

**Figure 1.**
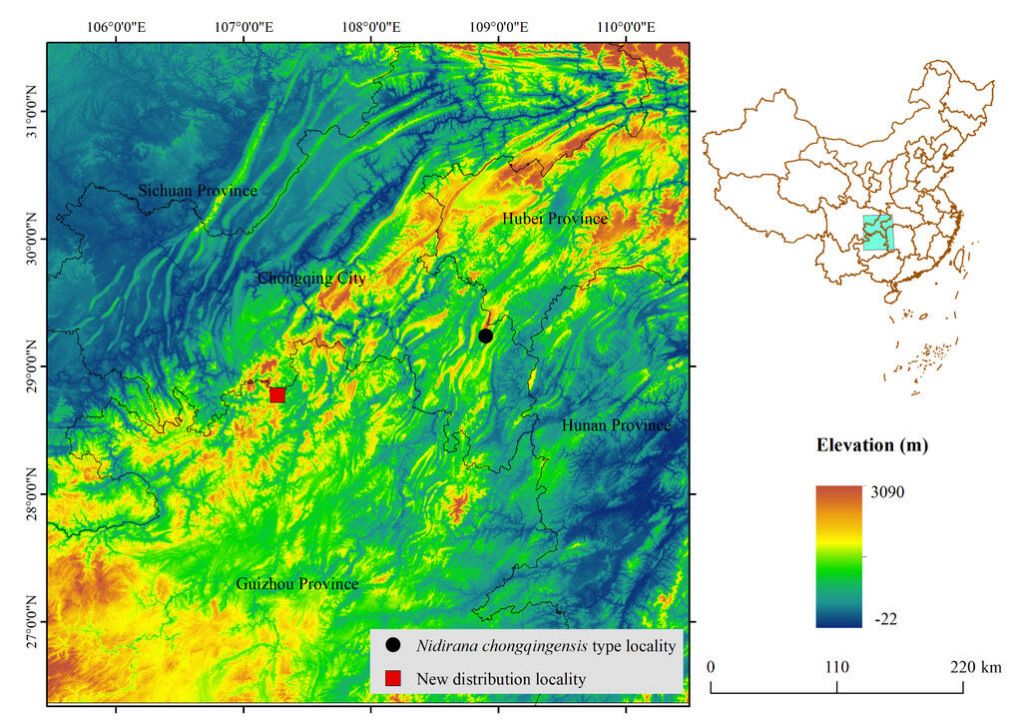
Distribution map of *Nidirana chongqingensis*.

Six adult specimens (3 males, 3 females) and five tadpoles were collected from montane ponds in Zheng'an County, Guizhou Province, China (28.77761° N, 107.26797° E, 1326 m a.s.l.; Fig. 1 on 7 July 2024 and 11 April 2025). Specimens were humanely euthanised using isoflurane, with fresh muscle tissue extracted and preserved in 100% ethanol prior to fixation. Adults were fixed in 75% ethanol, and tadpoles were preserved in 70% ethanol after photography. All specimens are deposited in the College of Resources and Environment, Moutai Institute (**MT**) (voucher numbers: MT ZA20240707001–002, 004-006, 008, MT ZA20250411007–011).

Sampling procedures complied with the Wild Animal Protection Law of China. The Animal Care and Use Committee of Guizhou University provided full approval for the research protocol (approval number: EAE-GZU-20244-T1228).

**Molecular phylogeny**

DNA was extracted from muscle tissue using a DNA extraction kit from Tiangen Biotech Co., Ltd. (Beijing, China). Two fragments of the mitochondrial 16S rRNA (16S) and cytochrome oxidase subunit I (COI) genes were amplified. For 16S, the primers P7 (5’-CGCCTGTTTACCAAAAACAT-3’) and P8 (5’-CCGGTCTGAACTCAGATCACGT-3’) were used (Simon et al. 1994), and for COI, Chmf4 (5’-TYTCWACWAAYCAYAAAGAYATCGG-3’) and Chmr4 (5’-ACYTCRGGRTGRCCRAARAATCA-3’) were used (Che et al. 2012). Gene fragments were amplified under the following conditions: an initial denaturing step at 95 °C for 4 min; 36 cycles of denaturing at 95 °C for 30 s, annealing at 52 °C (for 16S) or 47 °C (for COI) for 40 s, and extending at 72 °C for 70 s. Sequencing was conducted using an ABI3730 automated DNA sequencer by Shanghai DNA BioTechnologies Co., Ltd. (Shanghai, China). New sequences were deposited in GenBank (for GenBank accession numbers, see Table 1).

For phylogenetic analysis, 28 homologous sequences of *Nidirana* species and two outgroups (*Babina holsti*, *Babina subaspera*) were retrieved from GenBank (Table 1). Sequences were aligned using MUSCLE (Edgar 2004) in MEGA 6 (Tamura et al. 2013), and uncorrected p-distances were calculated. Maximum Likelihood (ML) trees were constructed on the CIPRES Science Gateway with 100 rapid bootstrap replicates (Miller et al. 2010), and Bayesian Inference (BI) was implemented in MrBayes 3.2 (Ronquist et al. 2012). BI analyses included four Markov chains run for 10 million generations, sampled every 500 generations, with the first 25% of samples discarded as burn-in, resulting in a potential scale reduction factor (PSRF) of < 0.005.

**Morphological measurements**

Adult morphological measurements were taken with a digital caliper (accuracy 0.1 mm) following Fei et al. (2009) and Ma and Wang (2023): snout-vent length (SVL), head length (HL), head width (HW), snout length (SL), internasal distance (IND), interorbital distance (IOD), upper eyelid width (UEW), eye diameter (ED), tympanum diameter (TD), length of lower arm and hand (LAHL), diameter of lower arm (LAD), hand length (HNL), hind-limb length (HLL), tibia length (TL), tibia width (TW), tarsus and foot length (LFT), and foot length (FL). Sex was determined by gonadal inspection and presence of nuptial pads/vocal sacs in males.

Tadpole measurements were conducted using ImageJ 1.54g (Schneider et al. 2012) from scaled photographs, following Fei et al. (2009) and Oberhummer et al. (2014): total length (TOL), snout-vent length (SVL), head length (HL), head width (HW), body length (BL), body height (BH), body width (BW), snout length (SL), snout to spiraculum (SS), tail length (TL), tail height, maximum tail height (TH), tail muscle width (TMW), interorbital distance (IOD), and mouth width (MW). Tadpole staging followed Gosner (1960), end external morphology terminology followed Altig and McDiarmid (1999).

## Data resources

The uncorrected p-distances of the COⅠ gene between the collected samples and *Nidirana chongqingensis* from its type locality were 0.0–0.4%, while the uncorrected genetic distances from other species of the genus *Nidirana* were 3.7–13.8% — which were much smaller than those between other congeneric species (Table 2).

For the 16S gene, the uncorrected p-distances between the collected samples and *Nidirana chongqingensis* from its type locality were 0.0–0.5%, and the uncorrected genetic distances from other *Nidirana* species were 1.0–7.7%, also much smaller than those between other congeneric species (Table 3).

**Figure 2.**
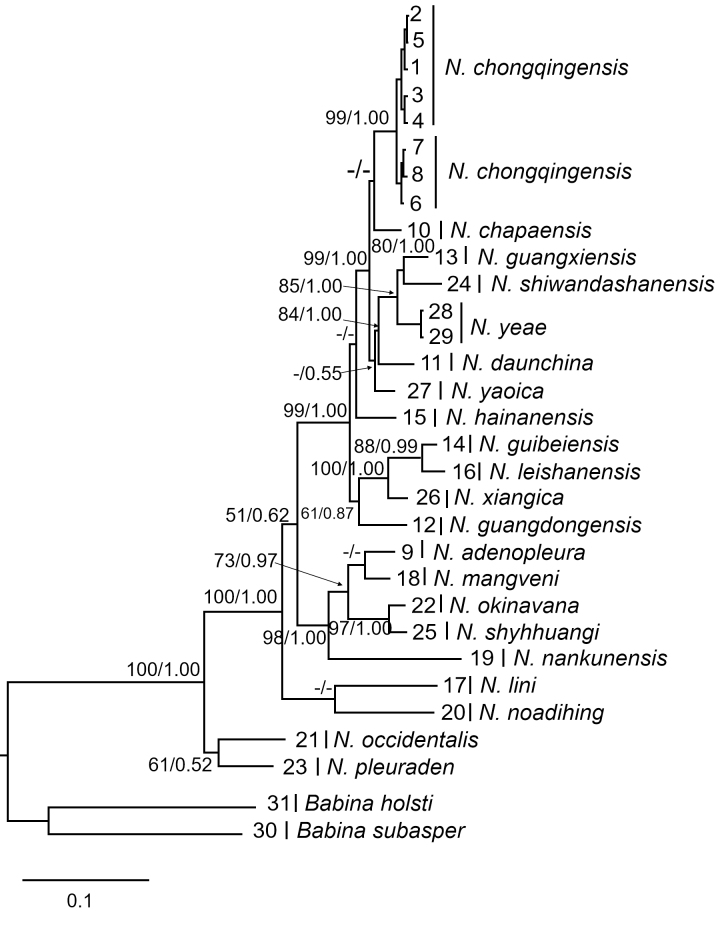
Reconstruction results of the Bayesian (Maximum Likelihood, ML) phylogenetic tree of *Nidirana*, based on 16S and CO1 genes: numbers on nodes represent branch support values; samples 1–5 are from the distribution site in Zheng'an, Guizhou, China; samples 6–8 are from the type locality of *Nidirana chongqingensis*.

The phylogenetic topology of the genus *Nidirana* reconstructed, based on the 16S and CO1 genes is shown in Fig. 2. The *Nidirana chongqingensis* samples collected from Zhen'an County clustered into a single clade with those from the type locality, with high support values (99/1.00). This phylogenetic tree included samples of other *Nidirana* species, with *Babina holsti* and *Babina subaspera* as outgroups.

## Taxon treatments

### Nidirana chongqingensis

Ma & Wang, 2023

C2D5DEF8-951A-5936-BFB0-7935FF5E30A8

#### Materials

- **Type status:** Other material. **Occurrence:** catalogNumber: MT ZA20240707002, MT ZA20240707004, MT ZA20240707008; recordedBy: Jing Liu; Jiantao Pan; individualCount: 3; sex: male; lifeStage: adult; occurrenceID: E947C0B1-C793-58A2-AA8E-FF2280ABEF35; **Taxon:** scientificName: *Nidirana chongqingensis*; order: Anura; family: Ranidae; genus: Nidirana; **Location:** country: China; stateProvince: Guizhou; county: Zheng'an; verbatimElevation: 1326 m; verbatimCoordinates: 28.77761° N, 107.26797° E; **Event:** eventDate: 07-07-2024; **Record Level:** basisOfRecord: PreservedSpecimen
- **Type status:** Other material. **Occurrence:** catalogNumber: MT ZA20240707001, MT ZA20240707005, MT ZA20240707006; recordedBy: Jing Liu; Junjian Zhou; individualCount: 3; sex: female; lifeStage: adult; occurrenceID: 33C09773-8B59-539F-9717-194367FA7A08; **Taxon:** scientificName: *Nidirana chongqingensis*; order: Anura; family: Ranidae; genus: Nidirana; **Location:** country: China; stateProvince: Guizhou; county: Zheng'an; verbatimElevation: 1326 m; verbatimCoordinates: 28.77761° N, 107.26797° E; **Event:** eventDate: 07-07-2024; **Record Level:** basisOfRecord: PreservedSpecimen
- **Type status:** Other material. **Occurrence:** catalogNumber: MT ZA20250411007–011; recordedBy: Jing Liu; Jiantao Pan; individualCount: 5; sex: indeterminate; lifeStage: tadpole; occurrenceID: BF058042-BDBC-537F-A832-8D60D1AC433B; **Taxon:** scientificName: *Nidirana chongqingensis*; order: Anura; family: Ranidae; genus: Nidirana; **Location:** country: China; stateProvince: Guizhou; county: Zheng'an; verbatimElevation: 1326 m; verbatimCoordinates: 28.77761° N, 107.26797° E; **Event:** eventDate: 11-04-2025; **Record Level:** basisOfRecord: PreservedSpecimen

#### Description

Males

**Figure 3.**
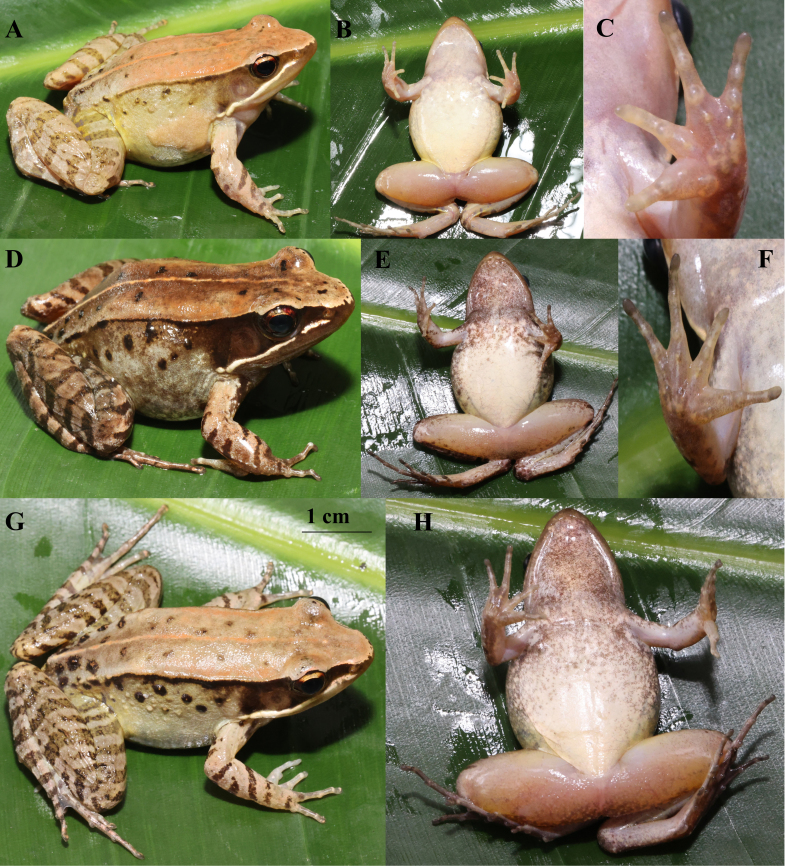
Live photographs of *Nidirana chongqingensis*: Male (specimen: MT ZA20240707008). **A** Lateral view; **B** Ventral view; **C** Ventral view of the hand; Female (specimen: MT ZA20240707005); **D** Lateral view; **E** Ventral view; **F** Ventral view of the hand; Female (specimen: MT ZA20240707006); **G** Lateral view; **H** Ventral view.

The morphological characteristics of the newly-collected male *Nidirana chongqingensis* specimens are mostly consistent with the diagnostic characters proposed by Ma and Wang (2023). The following description is based on three specimens (MT ZA20240707002, MT ZA20240707004, MT ZA20240707008): body size small (SVL 41.1-43.4 mm) ; indistinct canthus rostralis; supernumerary tubercles below the base of fingers III and IV, inconspicuous; well-developed dorsolateral folds, but intermittent posteriorly; males with large, smooth and protruding suprabrachial glands in breeding period; dorsal skin relatively smooth, without horny spines on the back; ventral surface of body milky-white; tibio-tarsal articulation reaching the angle of the eye or the nostril when adpressed along body, heels not meeting or just meeting when hind limbs flexed at a straight angle to the body's axis; males with one single nuptial pad on the dorsal surface of the first finger in the breeding period (Fig. 3, Table 4).

Males (based on MT ZA20240707008; Fig. 3, Table 4)

Morphology and colouration are mostly consistent with the description of Ma and Wang (2023). SVL 41.10–43.41 mm; a single nuptial pad is present on the dorsal base of Finger I; the ventral surface is milky-white, while females have dense brown spots on the throat, chest and ventral sides of the limbs; the possession of internal vocal sacs.

Females (based on MT ZA20240707005, female; Fig. 3, Table 4)

Body slender, SVL 45.15 mm; head longer than wide (HL/HW = 1.07); snout rounded in dorsal view, slightly protruding beyond lower jaw; loreal region slightly concave; canthus rostralis indistinct; pupil elliptical, horizontal; nostril rounded, directed laterally, closer to eye than snout (NEL/NSL = 0.98).

Dorsal skin relatively smooth, with scattered small warts on mid-dorsum; dorsolateral folds thin, distinct, extending from posterior corner of eye to groin (discontinuous posteriorly); supratympanic fold absent; tympanum distinct, TD/ED = 0.89.

Fore-limbs moderately robust; fingers thin, free of webbing, lateral fringes narrow; relative finger length: II < I < IV < III; subarticular tubercles rounded, prominent; supernumerary tubercles at base of fingers III–IV inconspicuous; metacarpal tubercles 3, elliptical.

Hind-limbs robust; tibia length 47.6% of SVL; tibiotarsal articulation reaching anterior corner of eye; heels not meeting when hind-limbs flexed at right angles to body axis; toes with moderate webbing (formula as above); toe tips slightly dilated; subarticular tubercles prominent; inner metatarsal tubercle oval, outer tubercle small and rounded.

Colouration in life: Dorsum brown with irregular dark brown spots; light brown mid-dorsal stripe from interorbital region to vent (no dark edges); flanks cream-yellow (upper) to light grey (lower); limbs with dark brown transverse bars; tympanum red-brown; iris upper 1/3 bright yellow, lower 2/3 brown-red. Ventral surface milky-white; throat, chest, and ventral limbs densely covered with brownish-red spots (darker in colour and denser than males); thigh ventral surface light yellow.

**Figure 4.**
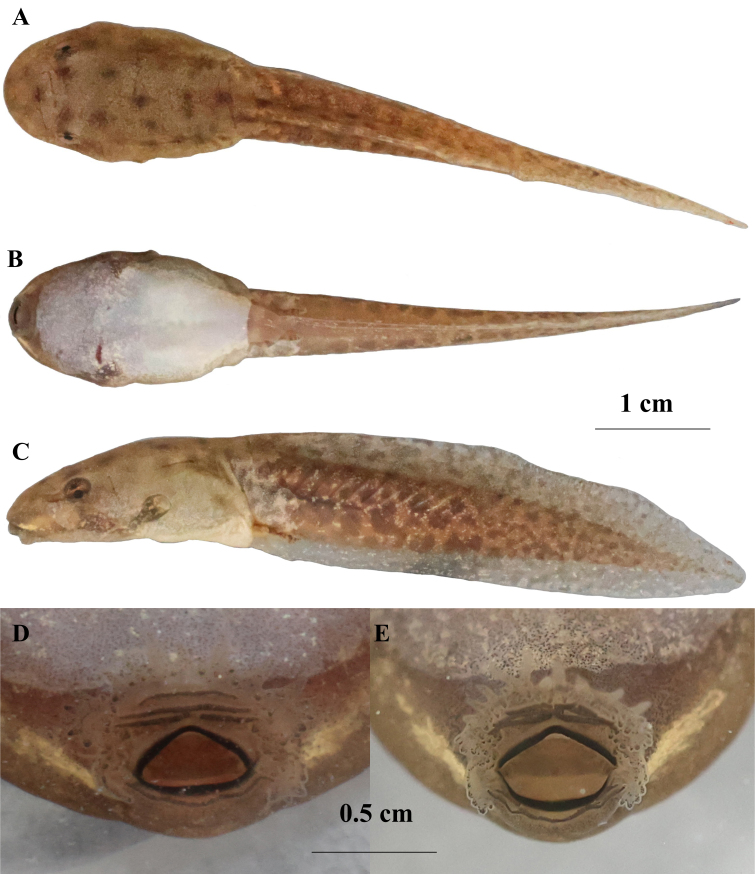
Stage 30 tadpole of *Nidirana chongqingensis* (Zheng'an, Guizhou, China). **A** Dorsal view; **B** Ventral view; **C** Lateral view; **D** Labial tooth row formula (specimen: MT ZA20250411010); **E** Labial tooth row formula (specimen: MT ZA20250411011).

Tadpoles (based on MT ZA20250411010, stage 30; Fig. 4, Table 5 .

Morphometrics: TOL 63.66 mm, SVL 21.48 mm, TL 41.73 mm (TL/TOL = 65.6%); body elongate-elliptical in dorsal view, flattened dorsoventrally (BH/BW = 0.70); eyes dorsolateral, not visible ventrally; pupils round; nostrils oval, directed laterally, closer to eye than snout (NED/SND = 1.19); narial rims slightly raised.

Spiracle single, sinistral, low on left body; spiracle tube short, free at tip, opening laterally (SSD/BL = 51.1%); anal tube medial, attached to ventral fin, opening posteriorly. Tail muscle strong, TMH/MTH = 65.2%; tail tip rounded; upper fin arising behind body-tail junction (UFH/MTH = 19.5%), lower fin connected to trunk (LFH/MTH = 23.9%).

Oral disc and mouthparts: Oral disc terminal, funnel-like, moderately sized. Upper jaw sheath wide, comb-like with a distinct median notch; lower jaw sheath thin, sickle-shaped, with fine serrations along the cutting edge.

Labial tooth row formula (LTRF): *I:1+1/1+1:II*. The upper labium bears 2 rows of oval submarginal papillae, with no papillae at the median notch of the upper jaw sheath; the lower labium has 3 rows of submarginal papillae, and small conical marginal papillae are present at the oral angles. Tooth rows are well-developed: the anterior upper row (Row I) is continuous, while the posterior upper rows (1+1) and lower rows (1+1:II) are clearly demarcated.

Colouration in life: Dorsal body yellowish-brown, scattered with irregular small brown spots (rather than diffuse pigments); tail muscle light yellow, with distinct brown spots clustered in the posterior section; tail fins semi-transparent, densely covered with fine brown speckles; ventral body and tail are translucent with a faint whitish tint, and the gut coil is barely visible; area around the eye is dotted with golden speckles.

#### Diagnosis

Revised diagnosis

*Nidirana chongqingensis* is distinguished from congeners by the following combination of characters:

- Small to medium body size (SVL 41.1–43.4 mm in males, 42.2–47.0 mm in females);
- Indistinct canthus rostralis; no longitudinal ridges on upper arms;
- Lateroventral grooves present on all fingers and toes;
- Males with a single nuptial pad on the dorsal base of Finger I during the breeding season;
- Males with internal vocal sacs;
- Males with a milky-white ventral surface; females with the throat and chest densely covered with brown spots (spots denser than those in males).
- Tadpoles (stage 30) with TOL 47.1–63.7 mm, BL 14.5–21.5 mm, TL accounting for 63.5–67.1% of TOL; oral disc funnel-like, upper jaw sheath with median notch;
- Webbing moderate, webbing formula: I 1/2 – 1 II 1/2 – 2 III 1 – 2½ IV 2 – 1V;
- Tibio-tarsal articulation reaching eye or nostril when adpressed.

#### Distribution

*Nidirana chongqingensis* is now known from two localities in south-western China:

Type locality: Gaolu Village, Mala Town, Qianjiang District, Chongqing Municipality, China (29.24203° N, 108.902202° E, 1419 m a.s.l.) (Ma and Wang 2023);

New locality: Montane ponds of Xinzhou Town, Zheng'an County, Guizhou Province, China (28.77761° N, 107.26797° E, 1326 m a.s.l.).

#### Ecology

**Figure 5.**
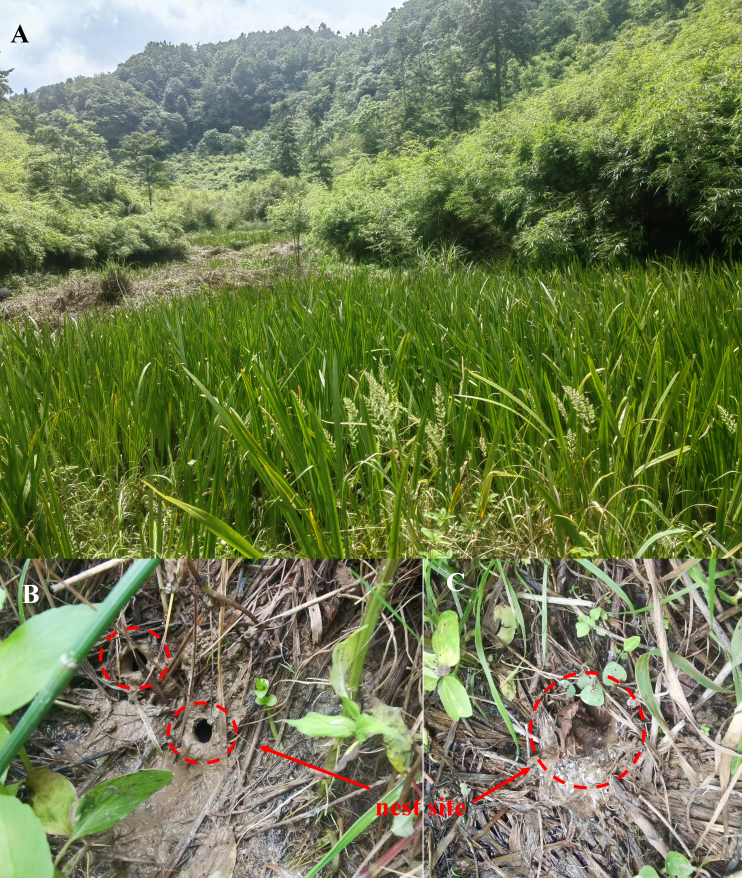
Habitat of *Nidirana chongqingensis* (Zheng'an, Guizhou, China). **A** Habitat; **B, C** Nests.

The habitat consists of montane swamps, ponds, and aquatic grasslands covered with dense weeds (Fig. 5A). Adults hide in thick weed patches; males call at night, and this species co-exists with sympatric taxa, including *Polypedates megacephalus*, *Zhangixalus chenfui* and *Hylarana guentheri*. In this habitat, *N. chongqingensis* has been observed to exhibit nesting behaviour, with the nests being circular in shape (Fig. 5 B and C). Tadpoles are found in stagnant pools within ponds, co-existing with other aquatic animals.

## Discussion

Our study confirms the presence of *N. chongqingensis* in Guizhou Province, extending its distribution range by approximately 300 km northwest of the type locality. Molecular analyses support this conclusion: new specimens cluster with *N. chongqingensis* type sequences (16S p-distance = 0.000–0.005; COI p-distance = 0.000–0.004) (Tables 4, 5), well below the interspecific divergence threshold for *Nidirana* (≥ 2.3%; Lyu et al. (2019), Ma and Wang (2023)).

The first description of females and tadpoles fills key gaps in the biology of *N. chongqingensis*. Females are slightly larger than males (SVL 42.2–47.0 mm vs. 41.1–43.4 mm) and have darker in colour and denser ventral spots, while tadpoles exhibit typical ranid features (funnel-like oral disc, sinistral spiracle) consistent with *Nidirana* larvae (Grosjean et al. 2015). Males from the Zheng'an population in Guizhou Province possess internal vocal sacs, a trait inconsistent with the original description of the type locality population. This discrepancy suggests the original account of vocal sac morphology for the type locality may have contained inaccuracies, and we herein revise this diagnostic character for *Nidirana chongqingensis*. A survey of vocal sacs across all species of the genus *Nidirana* shows that the vast majority of them have internal vocal sacs. Revised diagnostic characters incorporate sexual dimorphism and tadpole traits, improving species identification accuracy.

Habitat at the new locality is similar to the type locality (montane swamps with clean water), but faces potential threats from agricultural pollution and habitat fragmentation - ommon risks for amphibians in southwest China (Lyu et al. 2021). The IUCN Red List status of *N. chongqingensis* remains unassessed (IUCN 2022); given its restricted range and habitat vulnerability, future conservation efforts should focus on protecting pond and farmland ecosystems in both Chongqing and Guizhou.

## Supplementary Material

XML Treatment for Nidirana chongqingensis

## Figures and Tables

**Table 1.** Localities, voucher information and GenBank numbers for samples used in this study.

ID	Species	16S	COI	Sample No.	Locality	Reference
1	*Nidirana chongqingensis*	PX925747	PX929382	MT ZA20240707005	China:Guizhou:Zheng'an	This study
2	*N . chongqingensis*	PX925748	PX929383	MT ZA20240707006	China:Guizhou:Zheng'an	This study
3	*N . chongqingensis*	PX925749	PX929384	MT ZA20240707008	China:Guizhou:Zheng'an	This study
4	*N . chongqingensis*	PX925750	PX929385	MT ZA20250411010	China:Guizhou:Zheng'an	This study
5	*N . chongqingensis*	PX925751	PX929386	MT ZA20250411011	China:Guizhou:Zheng'an	This study
6	*N. chongqingensis*	OQ846777	OQ843905	SWU0001408	China: Chongqing:Qianjiang	Ma and Wang 2023
7	*N. chongqingensis*	OQ846779	OQ843907	SWU0001439	China: Chongqing:Qianjiang	Ma and Wang 2023
8	*N. chongqingensis*	OQ846778	OQ843906	SWU0001435	China: Chongqing:Qianjiang	Ma and Wang 2023
9	*N. adenopleura*	MN946445	MN945201	SYS a007358	China: Taiwan:Taichung City	Lyu et al. 2020a
10	*N. chapaensis*	KR827711	KR087625	MNHN 2000.4850	Vietnam: Lao Cai:Sapa	Grosjean et al. 2015
11	*N. daunchina*	MF807822	MF807861	SYS a004594	China: Sichuan: Mt Emei	Lyu et al. 2020a
12	*N. guangdongensis*	MN946406	MN945162	SYS a005767	China: Guangdong:Yingde City	Lyu et al. 2020a
13	*N. guangxiensis*	MZ677222	MZ678729	NHMG 202007001	China: Guangxi: Mt Daming	Lyu et al. 2021
14	*N. guibeiensis*	ON985180	ON968962	NNU 00917	China: Guangxi:Xing’an: Maoershan	Chen et al. 2022a
15	*N. hainanensis*	MN946451	MN945207	SYS a007669	China: Hainan: Mt Diaoluo	Lyu et al. 2020a
16	*N. leishanensis*	MN946453	MN945209	SYS a007908	China: Guizhou: MtLeigong	Li et al. 2019
17	*N. lini*	MF807818	MF807857	SYS a003967	China: Yunnan:Jiangcheng	Lyu et al. 2017
18	*N. mangveni*	MN946424	MN945180	SYS a006310	China: Zhejiang: Mt Dapan	Lyu et al. 2020a
19	*N. nankunensis*	MF807839	MF807878	SYS a005718	China: Guangdong: Mt Nankun	Lyu et al. 2017
20	*N. noadihing*	OR656453	OR652452	WII-ADA1765	India: Arunachal Pradesh, Changlang，	Boruah et al. 2023
21	*N. occidentalis*	MF807816	MF807855	SYS a003775	China: Yunnan: Mt Gaoligong	Lyu et al. 2020a
22	*N. okinavana*	NC022872	NC022872	Unknown	Japan: Okinawa,Iriomote Island	Kakehashi et al. 2013
23	*N. pleuraden*	MT935683	MT932858	SYS a007858	China: Yunnan:Kunming City	Lyu et al. 2020b
24	*N. shiwandashanensis*	MZ787977	MZ782098	NNU00238	China: Guangxi:Shangsi	Chen et al. 2022a
25	*N. shyhhuangi*	PQ367823	PQ358973	Lienhuachih06	China:Taiwan:Lienhuachih	Lin et al. 2025
26	*N. xiangica*	MN946434	MN945190	SYS a006492	China: Hunan: Mt Dawei	Lyu et al. 2020a
27	*N. yaoica*	MK882276	MK895041	SYS a007020	China: Guangxi: Mt Dayao	Lyu et al. 2019
28	*N. yeae*	MN295228	MN295234	CIBTZ20190608005	China: Guizhou:Tongzi	Wei et al. 2020
29	*N. yeae*	MN295231	MN295237	CIBTZ20160714016	China: Guizhou:Tongzi	Wei et al. 2020
30	*Babina subasper*	NC022871	NC022871	Unknown	Japan: Kagoshima:	Kakehashi et al. 2013
31	*Babina holsti*	NC022870	NC022870	Unknown	Japan: Okinawa	Kakehashi et al. 2013

**Table 2.** Uncorrected p-distances based on COI genes amongst all *Nidirana* species.

*Nidirana chongqingensis* (Guizhou)																					
*N. chongqingensis* (Chongqing)	0.004																				
*N. yaoica*	0.037	0.026																			
*N. chapaensis*	0.043	0.032	0.029																		
*N. yeae*	0.046	0.035	0.023	0.030																	
*N. shiwandashanensis*	0.051	0.040	0.039	0.043	0.027																
*N. guangxiensis*	0.052	0.042	0.030	0.034	0.018	0.027															
*N. hainanensis*	0.056	0.042	0.043	0.043	0.045	0.054	0.052														
*N. daunchina*	0.060	0.049	0.038	0.048	0.039	0.048	0.043	0.054													
*N. leishanensis*	0.067	0.056	0.055	0.055	0.059	0.061	0.055	0.055	0.063												
*N. hainanensis*	0.070	0.060	0.055	0.055	0.055	0.064	0.059	0.061	0.063	0.027											
*N. guangdongensis*	0.075	0.065	0.054	0.061	0.052	0.068	0.066	0.057	0.059	0.063	0.057										
*N. noadihing*	0.098	0.088	0.088	0.073	0.080	0.082	0.084	0.100	0.082	0.095	0.089	0.093									
*N. adenopleura*	0.099	0.089	0.075	0.082	0.082	0.091	0.086	0.084	0.089	0.096	0.091	0.089	0.080								
*N. occidentalis*	0.132	0.124	0.118	0.111	0.121	0.114	0.125	0.118	0.113	0.132	0.129	0.127	0.113	0.113							
*N. xiangica*	0.072	0.061	0.057	0.054	0.057	0.073	0.061	0.057	0.064	0.030	0.029	0.064	0.100	0.105	0.129						
*N. lini*	0.104	0.095	0.096	0.086	0.086	0.096	0.096	0.095	0.093	0.111	0.102	0.096	0.077	0.096	0.114	0.109					
*N. nankunensis*	0.110	0.101	0.100	0.096	0.096	0.113	0.104	0.105	0.109	0.098	0.098	0.091	0.100	0.071	0.125	0.102	0.093				
*N. mangveni*	0.110	0.099	0.091	0.093	0.095	0.104	0.100	0.095	0.096	0.107	0.102	0.098	0.084	0.048	0.113	0.109	0.098	0.070			
*N. okinavana*	0.112	0.101	0.089	0.086	0.089	0.096	0.089	0.095	0.095	0.107	0.102	0.093	0.088	0.046	0.114	0.111	0.104	0.071	0.048		
*N. shyhhuangi*	0.117	0.108	0.095	0.091	0.095	0.102	0.095	0.102	0.100	0.109	0.100	0.095	0.089	0.048	0.114	0.109	0.105	0.077	0.050	0.027	
*N. pleuraden*	0.138	0.129	0.123	0.125	0.118	0.120	0.129	0.120	0.121	0.145	0.145	0.116	0.121	0.109	0.086	0.143	0.111	0.116	0.127	0.111	0.111

Note: Samples of *Nidirana chongqingensis* were collected from Zheng'an, Guizhou, China; samples of *N. chongqingensis* were obtained from the type locality (Chongqing, China).

**Table 3.** Uncorrected p-distances based on 16S genes amongst all *Nidirana* species.

*Nidirana chongqingensis* (Guizhou)																					
*N. chongqingensis* (Chongqing)	0.005																				
*N. guangxiensis*	0.010	0.003																			
*N. yeae*	0.015	0.008	0.005																		
*N. shiwandashanensis*	0.018	0.010	0.008	0.013																	
*N. yaoica*	0.020	0.013	0.010	0.016	0.018																
*N. chapaensis*	0.020	0.013	0.010	0.016	0.013	0.021															
*N. daunchina*	0.020	0.013	0.010	0.016	0.018	0.021	0.021														
*N. hainanensis*	0.022	0.021	0.021	0.026	0.029	0.031	0.031	0.031													
*N. xiangica*	0.023	0.021	0.018	0.023	0.026	0.029	0.029	0.029	0.029												
*N. guangdongensis*	0.028	0.026	0.023	0.029	0.031	0.034	0.034	0.034	0.029	0.029											
*N. guibeiensis*	0.028	0.026	0.023	0.029	0.031	0.034	0.034	0.034	0.029	0.016	0.034										
*N. lini*	0.031	0.029	0.026	0.026	0.034	0.037	0.037	0.037	0.037	0.034	0.039	0.034									
*N. leishanensis*	0.035	0.034	0.034	0.039	0.042	0.039	0.044	0.044	0.034	0.026	0.044	0.010	0.044								
*N. noadihing*	0.039	0.037	0.037	0.037	0.044	0.047	0.047	0.047	0.042	0.039	0.050	0.044	0.021	0.050							
*N. okinavana*	0.041	0.039	0.037	0.037	0.044	0.047	0.042	0.047	0.042	0.044	0.050	0.034	0.031	0.044	0.037						
*N. adenopleura*	0.041	0.039	0.037	0.037	0.044	0.047	0.042	0.047	0.042	0.039	0.047	0.029	0.037	0.039	0.042	0.010					
*N. mangveni*	0.041	0.039	0.037	0.037	0.044	0.047	0.042	0.047	0.042	0.042	0.047	0.031	0.031	0.042	0.037	0.005	0.005				
*N. shyhhuangi*	0.043	0.042	0.039	0.039	0.047	0.050	0.044	0.050	0.044	0.042	0.052	0.037	0.034	0.047	0.039	0.003	0.013	0.008			
*N. occidentalis*	0.062	0.060	0.057	0.052	0.063	0.068	0.065	0.060	0.068	0.060	0.065	0.068	0.052	0.078	0.047	0.057	0.060	0.057	0.060		
*N. pleuraden*	0.067	0.060	0.057	0.057	0.065	0.063	0.063	0.060	0.068	0.055	0.076	0.063	0.055	0.073	0.050	0.055	0.060	0.055	0.052	0.042	
*N. nankunensis*	0.077	0.076	0.073	0.068	0.081	0.084	0.078	0.084	0.073	0.081	0.086	0.070	0.068	0.076	0.070	0.047	0.052	0.047	0.050	0.084	0.084

Note: Samples of *Nidirana chongqingensis* were collected from Zheng'an, Guizhou, China; samples of *N. chongqingensis* were obtained from the type locality (Chongqing, China).

**Table 4.** Measurements of adult specimens of *Nidirana chongqingensis* from (in mm) from Zheng'an, Guizhou, China.

Voucher No.	MT ZA20240707001	MT ZA20240707005	MT ZA20240707006	MT ZA20240707002	MT ZA20240707004	MT ZA20240707008
Sex	Female	Female	Female	Male	Male	Male
SVL	45.15	42.25	47.03	43.41	41.28	41.1
HL	14.7	14.56	15.62	14.36	14.97	14.22
HW	13.72	15.01	15.27	13.53	13.59	13.51
SL	6.7	6.94	7.69	7.51	7.01	6.91
IND	5.78	5.54	5.72	5.44	5.18	5.76
IOD	4.35	3.99	4.57	5.33	4.75	4.61
UEW	3.82	3.7	3.13	3.67	3.5	3.23
ED	4.57	4.65	3.93	4.52	4.6	4.43
TD	4.08	3.65	4.49	4.6	3.67	3.86
LAHL	19.61	19.67	20.86	19.38	19.15	18.34
LAD	2.93	3.23	3.19	3.2	3.42	3.62
HNL	11.69	11.16	12.74	11.66	11.26	11.28
HLL	73.46	74.24	76.36	72.92	68.76	67.45
TL	24.19	23.67	24.54	23.18	22.36	20.87
TW	5.58	5.65	6.26	5.72	5.17	5.19
LFT	34.34	34.47	37.69	34.25	32.79	31.52
FL	21.45	21.86	24.77	22.87	22.59	20.89

**Table 5.** Measurements of *Nidirana chongqingensis* tadpoles (in mm) from Zheng'an, Guizhou, China.

**Voucher**	MT ZA20250411007	MT ZA20250411008	MT ZA20250411009	MT ZA20250411010	MT ZA20250411011
**Stage**	30	30	30	30	30
**TOL**	63.18	63.66	61.21	51.33	47.09
**SVL**	20.36	21.48	19.24	17.09	14.48
**HL**	9.29	9.9	8.33	7.76	7.49
**HW**	8.44	9.28	8.29	9.39	7.91
**BH**	6.15	7.02	6.43	6.25	5.43
**BW**	9.7	9.98	9.91	9.62	9.21
**SL**	6.08	5.22	5.71	5.39	4.58
**SS**	12.08	10.98	11.18	10.83	10.37
**TH**	8.24	10.59	9.96	7.48	7.47
**TL**	42.58	41.73	38.75	32.38	29.94
**TMW**	5.11	4.6	4.96	4.42	3.69
**IOD**	6.31	4.48	5.12	3.94	4.04
**MW**	4.18	4.73	3.9	3.9	3.93
